# A new era in the management of diabetic kidney disease

**DOI:** 10.5339/qmj.2023.26

**Published:** 2024-01-17

**Authors:** Mohamad M. Alkadi

**Affiliations:** 1 Division of Nephrology, Department of Medicine, Hamad General Hospital, Doha, Qatar Email: malkadi@hamad.qa; 2 Weill Cornell Medicine, Doha, Qatar

Kidney disease is currently one of the top ten causes of death globally, with cardiovascular disease being the leading cause of death among patients with kidney diseases.^1,2^ Diabetic kidney disease (DKD) is the most common form of chronic kidney disease (CKD), accounting for up to 40% of cases. Since the early 1990s, angiotensin-converting enzyme inhibitors (ACEI) and angiotensin II receptor blockers (ARBs) have been the mainstay of treatment for DKD patients, given their proven renoprotective, cardioprotective, and antiproteinuric effects.^3,4^ However, physicians did not have much to offer patients once they maximized the doses of these medications other than controlling their blood pressure and blood sugar and applying lifestyle modifications.^5^

From 2016 onwards, numerous studies showed that a new class of antidiabetic medications, known as sodium-glucose transport protein 2 inhibitors (SGLT2i), exhibits a synergistic effect with ACEI/ARBs in terms of renal and cardiac outcomes in DKD patients.^6-11^ All these studies were multicenter, randomized, double-blind, placebo-controlled trials.

In 2019, Canagliflozin became the first SGLT2i to receive the U.S. Food and Drug Administration (FDA) approval for treating DKD patients with an estimated glomerular filtration rate (eGFR) between 30-90 mL/minute/1.73 m^2^. In 2020, DAPA-CKD, a landmark study in managing CKD patients, was published [10]. It was the first study to assess the efficacy and safety of the SGLT2i, Dapagliflozin, in adult CKD patients with eGFR as low as 25 mL/minute/1.73 m^2^, with or without type 2 diabetes mellitus (T2DM).

Compared to placebo, the use of Dapagliflozin resulted in a significant relative risk reduction (39%) of a composite primary outcome of decline in eGFR ≥ 50%, end-stage kidney disease (ESKD), and death from renal or cardiovascular causes in both diabetic and non-diabetic patients.

Most recently, the results of the EMPA-KIDNEY study, which assessed the efficacy and safety of the SGLT2i, Empagliflozin, in adult CKD patients with eGFR as low as 20 mL/minute/1.73 m^2^, with or without T2DM, were published.^11^ Using Empagliflozin resulted in a significant relative risk reduction (28%) of a composite primary outcome of ESKD, a sustained decrease in eGFR to <10 mL/minute/1.73 m^2^, a sustained reduction in eGFR of ≥ 40% from baseline, or death from renal or cardiovascular causes.

In late 2020, a new nonsteroidal, selective mineralocorticoid receptor antagonist (MRA) called Finerenone was shown to improve renal and cardiac outcomes in DKD patients synergistically with ACEI/ARBs.^12^ The FIDELIO-DKD study included adult DKD patients with an eGFR as low as 25 ml per minute per 1.73 m^2^ and moderately to severely increased albuminuria. The use of Finerenone in such patients resulted in a significant risk reduction rate (18%) of a primary composite outcome of kidney failure, a sustained decrease in eGFR of ≥40% from baseline, or death from renal causes. It also resulted in a significant risk reduction rate (14%) for a secondary composite outcome of death from cardiovascular causes, non-fatal myocardial infarction, non-fatal stroke, or hospitalization for heart failure compared to placebo.

Given the overwhelming evidence of their renal and cardiac efficacy and safety, Both SGLT2i and nonsteroidal MRA are now part of the Kidney Disease Improving Global Outcomes (KDIGO) and the American Diabetes Association (ADA) guidelines.^13,14^ These guidelines recommend using SGLT2i as a first-line therapy in DKD patients with eGFR ≥ 20 mL/min/1.73 m^2^ with or without albuminuria. They also recommend using nonsteroidal MRA (Finerenone) in DKD patients with eGFR ≥ 25 mL/min/1.73 m^2^ and moderately to severely increased albuminuria. Thus, in the current era physicians have more treatment options to manage DKD patients leading to better long-term renal and cardiac outcomes.
